# HNRNPH2-R114W remodels RNA splicing and neurodevelopmental gene programs in isogenic cerebral organoids

**DOI:** 10.3389/fbioe.2026.1922305

**Published:** 2026-10-16

**Authors:** Carlo Donato Caiaffa, Neda Ghousifam, Stephan Lloyd Watkins, Helder I. Nakaya, Rodney A. Bowling, Richard H. Finnell, Robert M. Cabrera

**Affiliations:** 1 Dell Pediatric Research Institute, University of Texas at Austin, Austin, TX, United States; 2 Computational Systems Biology Laboratory, Universidade de São Paulo, São Paulo, Brazil; 3 Instituto do Cérebro, Universidade Federal do Rio Grande do Norte, Natal, Brazil; 4 To Cure a Rose Foundation, Austin Community College, Austin, TX, United States; 5 Hospital Israelita Albert Einstein, São Paulo, Brazil; 6 Institut Pasteur de São Paulo, São Paulo, Brazil; 7 Department of Clinical and Toxicological Analyses, School of Pharmaceutical Sciences, University of São Paulo, São Paulo, Brazil; 8 AlphaRose Therapeutics, Houston, TX, United States; 9 Baylor College of Medicine, Center for Precision Environmental Health, Houston, TX, United States

**Keywords:** alternative splicing, autism spectrum disorder, cerebral organoids, CRISPR correction, HNRNPH2, neurodevelopmental disorders, RNA-binding proteins, RNA-seq

## Abstract

Autism spectrum disorder has been linked to disrupted RNA processing during corticogenesis, yet variant-specific mechanisms remain difficult to resolve. Isogenic cerebral organoids derived from a patient-derived induced pluripotent stem cell (iPSC) line carrying the HNRNPH2-R114W variant and its CRISPR-corrected counterpart serve as a human developmental model to investigate variant-associated RNA dysregulation. Imaging quantification and exploratory bulk RNA sequencing, performed at a single developmental time point on a pooled organoid RNA from each genotype, identify coordinated remodeling of neuronal and metabolic programs, along with junction-centered splicing dysregulation involving 6,310 differentially used junctions. Computational structural docking situates *HNRNPH2* RNA recognition motifs (RRMs) 1–3 within a spliceosomal context and locates residue R114 near a predicted RNA-contact interface, indicating that the R114W substitution may alter RNA engagement. Integration of differential expression with event- and isoform-level analyses identifies regulatory hubs enriched for axon guidance and synaptic signaling pathways. Single-cell-reference-based deconvolution suggests modest compositional shifts that are unlikely to account for the extent of transcriptional and splicing alterations observed. Collectively, these findings imply HNRNPH2-R114W in widespread RNA rewiring and define candidate isoform-level endpoints for mechanistic and therapeutic investigation in human cortical organoids. These molecular signatures open new avenues to examine how HNRNPH2-associated RNA dysregulation evolves across developmental time and recurs across independent biological contexts, providing a foundation for further mechanistic studies of this rare neurodevelopmental disorder.

## 1 Introduction

Autism spectrum disorder (ASD) and related neurodevelopmental disorders (NDDs) arise from diverse genetic and environmental factors that collectively influence early cortical development. Recent human transcriptomic studies demonstrate that RNA processing, particularly alternative splicing, is a critical contributor to ASD and NDD risk, as dynamic splicing programs regulate corticogenesis, neuronal subtype specification, synaptic maturation, and circuit function (Raj and Blencowe, 2015; Li et al., 2016; Weyn-Vanhentenryck et al., 2018; Irimia et al., 2014). These findings indicate that RNA dysregulation in ASD cannot be fully explained by gene-level expression alone, necessitating analytical approaches that also consider transcript-, exon-, and splice-junction-level structures.

In the developing brain, splicing regulation functions as a coordinated developmental network responsible for generating neuron-specific isoforms, modulating neuronal excitability, and shaping synaptic signaling during defined developmental periods (Raj and Blencowe, 2015; Ule and Blencowe, 2019). During fetal corticogenesis, RNA-binding proteins (RBPs) such as RBFOX, NOVA, and heterogeneous nuclear ribonucleoproteins (HNRNPs) orchestrate precisely timed splicing transitions. Disruption of these networks predominantly affects synaptic and projection-related gene programs, which are enriched in ASD and NDDs (Li et al., 2016; Weyn-Vanhentenryck et al., 2018; Huelga et al., 2012; Irimia et al., 2014). Specifically, HNRNP H-family proteins regulate splice site selection and prevent aberrant junctions at G-rich motifs, suggesting that their disruption leads to a junction-focused reorganization of neuronal isoform programs rather than merely the gain or loss of exons (Ule and Blencowe, 2019; Huelga et al., 2012; Honoré et al., 1995; Caputi and Zahler, 2001).

Rare pathogenic variants in *HNRNPH2* directly impact the RNA-processing machinery, resulting in an X-linked neurodevelopmental syndrome characterized by developmental delay, seizures, and a high prevalence of autistic traits (Bain et al., 2016; Pilch et al., 2018; Gillentine et al., 2021). *HNRNPH2* encodes a protein within the HNRNP H family that contains quasi-RNA recognition motifs and a PY-type nuclear localization signal, facilitating binding to G-rich RNA and nuclear import via the karyopherin-β2 pathway (Honoré et al., 1995; Caputi and Zahler, 2001; Brownmiller and Caplen, 2023). Disease-associated missense variants are located in the PY-NLS and qRRM domains and are predicted to impair nuclear trafficking or alter RNA binding specificity, leading to changes in splice-site selection and junction usage at HNRNP H/F target motifs (Huelga et al., 2012; Caputi and Zahler, 2001; Brownmiller and Caplen, 2023). Additional evidence from patient and family studies supports the concept of HNRNP-related “RBPopathy,” where disruption of RBP networks produces overlapping ASD/ID phenotypes, extensive alternative splicing, deregulation of neurodevelopmental genes, and potentially dominant-negative or toxic gain-of-function mechanisms, with only limited compensation by HNRNPH1 (Raj and Blencowe, 2015; Irimia et al., 2014; Gillentine et al., 2021; Brownmiller and Caplen, 2023; Larizza et al., 2022).

Brain organoids derived from human induced pluripotent stem cells (iPSCs) recapitulate key aspects of early corticogenesis and provide a robust platform for modeling monogenic neurodevelopmental disorders (NDDs), including those resulting from defects in RNA-processing genes (Lancaster et al., 2013; Paşca et al., 2015; Mariani et al., 2015; Quadrato et al., 2017; Bershteyn et al., 2017; Velasco et al., 2019). For splicing regulators and HNRNP proteins, organoids offer relevant cell types and developmental timelines, enabling the interpretation of gene-level expression changes alongside splicing outcomes at the junction and isoform levels. Furthermore, organoid-based ASD models reveal that risk variants can influence progenitor dynamics, neuronal differentiation, and early neural network formation even in the absence of overt morphological abnormalities. This observation aligns with the features of HNRNPH2-related NDDs and supports the hypothesis that ASD associated with HNRNPH2 arises from a spliceopathy mediated by RNA-binding proteins during cortical development (Mariani et al., 2015; Quadrato et al., 2017; Bershteyn et al., 2017; Raj and Blencowe, 2015; Irimia et al., 2014; Brownmiller and Caplen, 2023).

Variation in maturation state and cell-type composition across organoid lines and batches can complicate the interpretation of regulatory signatures in bulk organoid transcriptomes (Quadrato et al., 2017; Velasco et al., 2019; Qian et al., 2016). Application of deconvolution methods, such as multi-subject single-cell deconvolution (MuSiC) combined with a human neural organoid single-cell atlas (HNOCA), enables estimation of cell-type proportions and distinction of regulatory effects from lineage balance changes (Wang et al., 2019; He et al., 2024; Nowakowski et al., 2017; Pollen et al., 2019). Additionally, genome-edited isogenic pairs facilitate causal inference by comparing a patient-derived HNRNPH2-R114W line with its corrected counterpart within the same genetic background, thereby reducing inter-individual variability and enhancing detection of genotype-associated changes in gene expression, splice-junction usage, and inferred cell-type composition (Bershteyn et al., 2017; Velasco et al., 2019; Qian et al., 2016; He et al., 2024).

Considering this context, a multi-layer RNA-centric approach is necessary to elucidate how pathogenic *HNRNPH2* variants alter human cortical transcriptomes. Gene-level differential expression analysis reveals genotype-associated transcriptional remodeling in isogenic cerebral organoids, while junction- and feature-centric methods capture splice-junction architecture and isoform rebalancing that may not be apparent at the exon or gene level (Robinson et al., 2010; Love et al., 2014; Pervouchine et al., 2013; Goldstein et al., 2016).

The ultra-rare disorder associated with HNRNPH2, referred to as Bain type X-linked syndromic intellectual developmental disorder, results from pathogenic variants that cause developmental delay or intellectual disability, severe motor and language deficits, seizures, and autistic features (Bain et al., 2016; Gillentine et al., 2021; Brownmiller and Caplen, 2023). The individual described in this study possesses a *de novo* heterozygous HNRNPH2-R114W missense variant and exhibits severe neurodevelopmental impairment, including absence of verbal communication, impaired ambulation, seizures, and autism spectrum features. Despite strong clinical and mechanistic evidence implicating HNRNPH2 in NDD and ASD, the causal pathway linking HNRNPH2 variation to RNA phenotypes in the human cortex remains incompletely defined in tissue-relevant contexts where splicing programs and RBP networks are active (Lancaster et al., 2013; Velasco et al., 2019; Qian et al., 2016; Wang et al., 2019). In this study, isogenic HNRNPH2-R114W and CRISPR-corrected cerebral organoids provide a neurodevelopmental system for measuring gene-level expression, junction- and isoform-level splicing, and inferred cell-type composition within a genetically matched background. This investigation integrates these data layers to demonstrate how the HNRNPH2-R114W variant alters human cortical transcriptomes, delineates the junction-dominant spliceopathy and its relationship to differential expression, and identifies isoform-level endpoints that may inform therapeutic strategies for ASD cases associated with RBP dysregulation.

## 2 Results

### 2.1 Validation of iPSC lines and assessment of organoid development in the context of the HNRNPH2-R114W rare variant

To assess the functional effects of the HNRNPH2-R114W mutation during early neuronal development, cerebral organoids were generated from patient-derived induced pluripotent stem cell (iPSC) lines. One line carried the rare HNRNPH2-R114W variant (designated Minus), while the other was a CRISPR-corrected isogenic counterpart (designated Plus) (Figure 1A). DNA-level Sanger sequencing revealed sequence changes associated with CRISPR-mediated correction in positive induced pluripotent stem cell (iPSC) clones compared with the parental line. These results confirm the successful generation of the corrected isogenic Plus line (Supplementary Figure S1). Both Minus and Plus iPSC lines exhibited strong nuclear expression of POU5F1 (OCT4) and NANOG (Figure 1B; Supplementary Figures S2A,B), indicating maintenance of pluripotency under standard culture conditions. Immunofluorescence targeting HNRNPH2 revealed a moderate decrease in signal in Minus cells, whereas SHANK1 displayed a slight increase and greater variability (Figure 1B; Supplementary Figures S2E,F).

**FIGURE 1:**
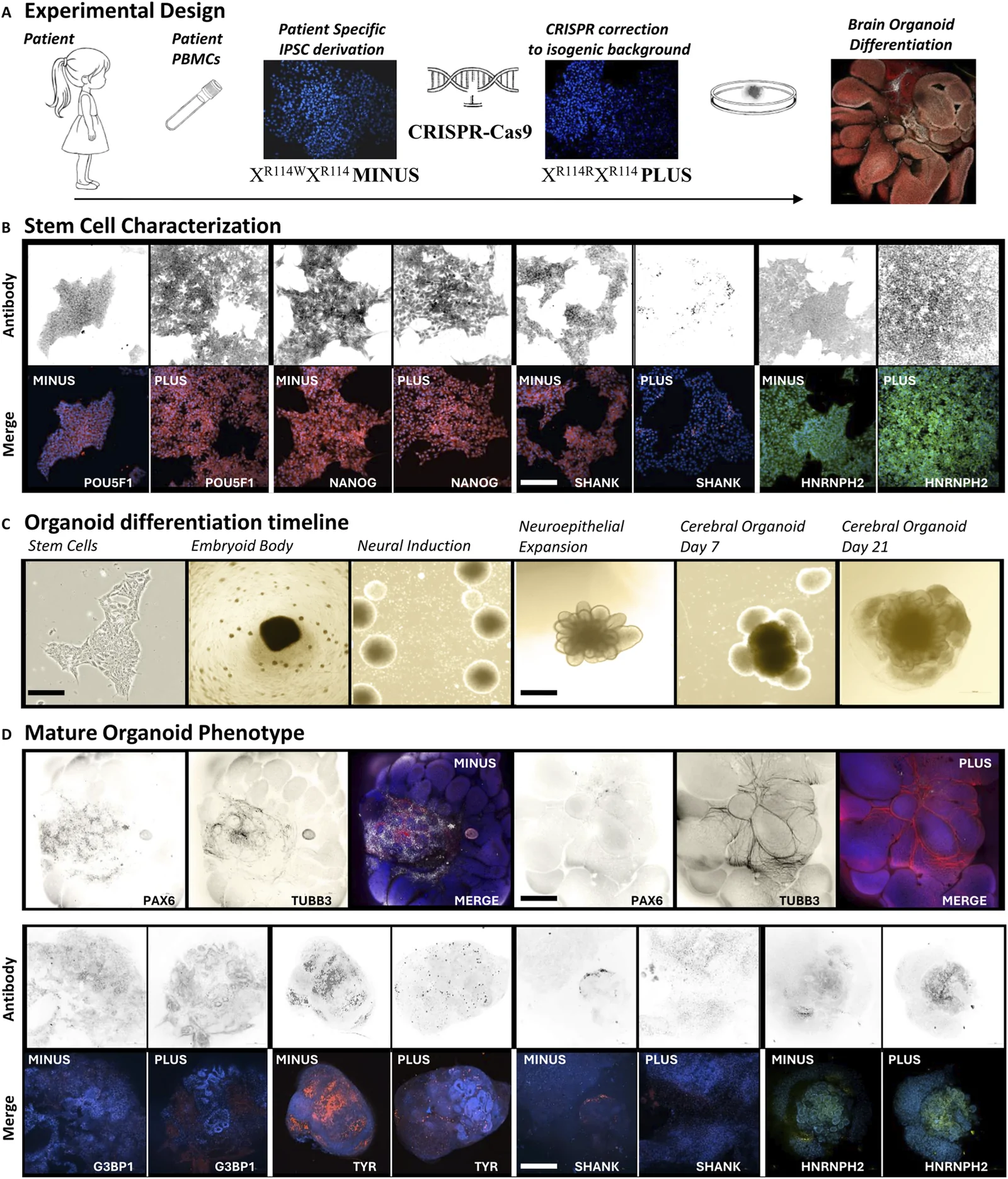
Characterization of stem cells and cerebral organoid differentiation establishes an isogenic HNRNPH2-R114W model that maintains early neurodevelopmental architecture. **(A)** Schematic overview of the experimental workflow, illustrating the generation of patient PBMC-derived iPSC lines harboring the HNRNPH2 R114W variant (“Minus”) and the CRISPR-corrected isogenic control (“Plus”), followed by differentiation into cerebral organoids. **(B)** Immunofluorescence analysis verifies pluripotency in both Minus and Plus iPSCs using nuclear POU5F1 (OCT4) and NANOG, alongside parallel staining for HNRNPH2 and SHANK1. The scale bar represents 200 µm. **(C)** Timeline schematic of the cerebral organoid protocol, detailing progression from stem cells through embryoid body formation, neural induction, and organoid maturation, with representative brightfield images at key stages, including days 7 and 21. The scale bar represents 100 and 200 μm, respectively. **(D)** Representative immunostaining of organoids demonstrates neuroepithelial and neuronal markers (PAX6 and TUBB3) with merged views, as well as additional marker panels comparing Minus and Plus organoids. The scale bar represents 100 µm. Images are representative of a minimum of three independently imaged organoids per condition across two differentiation batches. Selected single-channel panels are displayed as inverted black-and-white images to improve visualization of marker distribution and tissue morphology.

The differentiation protocol for cerebral organoids followed a standard timeline, including embryoid body formation, neural induction, neuroepithelial cell expansion, and early organoid maturation. At each stage, both Minus and Plus lineages generated neuroepithelial structures with a central lumen and radially arranged progenitor zones, resulting in larger, laminated neuroepithelial areas without observable morphological abnormalities. Organoids from both genotypes exhibited comparable size and general morphology at corresponding time points, with no apparent differences (Figures 1C,D).

Whole-mount immunostaining confirmed expression of canonical neurodevelopmental markers. PAX6-positive cells were organized in radial, ventricular-like zones surrounding the central lumen, while TUBB3-positive neurons localized to peripheral regions in both genotypes. Merged channel analysis demonstrated interconnected distribution of PAX6 and TUBB3, consistent with organoid maturation. No significant reduction in PAX6 domains or loss of TUBB3-positive neurons was observed in Minus compared to Plus organoids, indicating preservation of neuroepithelial and neuronal identities (Figure 1D; Supplementary Figures S2G,H).

### 2.2 HNRNPH2-directed immunofluorescence reveals genotype-dependent staining patterns

HNRNPH2-directed immunofluorescence revealed genotype-dependent staining patterns at the protein-signal level (Figure 1D; Supplementary Figure S2I). In Plus organoids, HNRNPH2 signal was predominantly localized to neuroepithelial regions, whereas Minus organoids exhibited weaker and more diffuse staining, suggesting altered distribution or cellular localization associated with the mutation. As this assay relied solely on immunofluorescence without orthogonal protein quantification, these findings are interpreted as changes in HNRNPH2 staining rather than definitive evidence of reduced protein abundance. By day 30, G3BP1 identified stress-responsive RNA granule structures, and TYR-positive cells marked pigmented regions enriched in the Minus lineage, consistent with melanocyte-like populations observed in subsequent RNA analyses. SHANK1 labeled synaptic candidate regions in both genotypes, with a stronger signal in Plus organoids (Supplementary Figures S2J–L). Collectively, these results validate the differentiation process in human iPSC-derived organoids and support their suitability for transcriptomic analysis by correlating morphology and marker expression with downstream molecular measurements.

### 2.3 Global transcriptional remodeling in HNRNPH2-R114W organoids

Gene expression patterns in Minus and Plus organoids were analyzed using a DESeq2 pipeline (Figure 2A). Pearson correlation analysis demonstrated high intra-group similarity and clear inter-group distinction (Figure 2B). Ordination analyses revealed strong separation between Minus and Plus samples along principal component 1 (PC1), which accounted for 99.5% of the variance, while PC2 accounted for 0.3% (Figure 2C; Supplementary Figure S3B). Given the rarity of such a dominant first principal component in bulk RNA-seq datasets, this result is interpreted as evidence of strong separation between genotype-specific pooled transcriptomes, rather than as a direct measure of variance among biological replicates.

**FIGURE 2:**
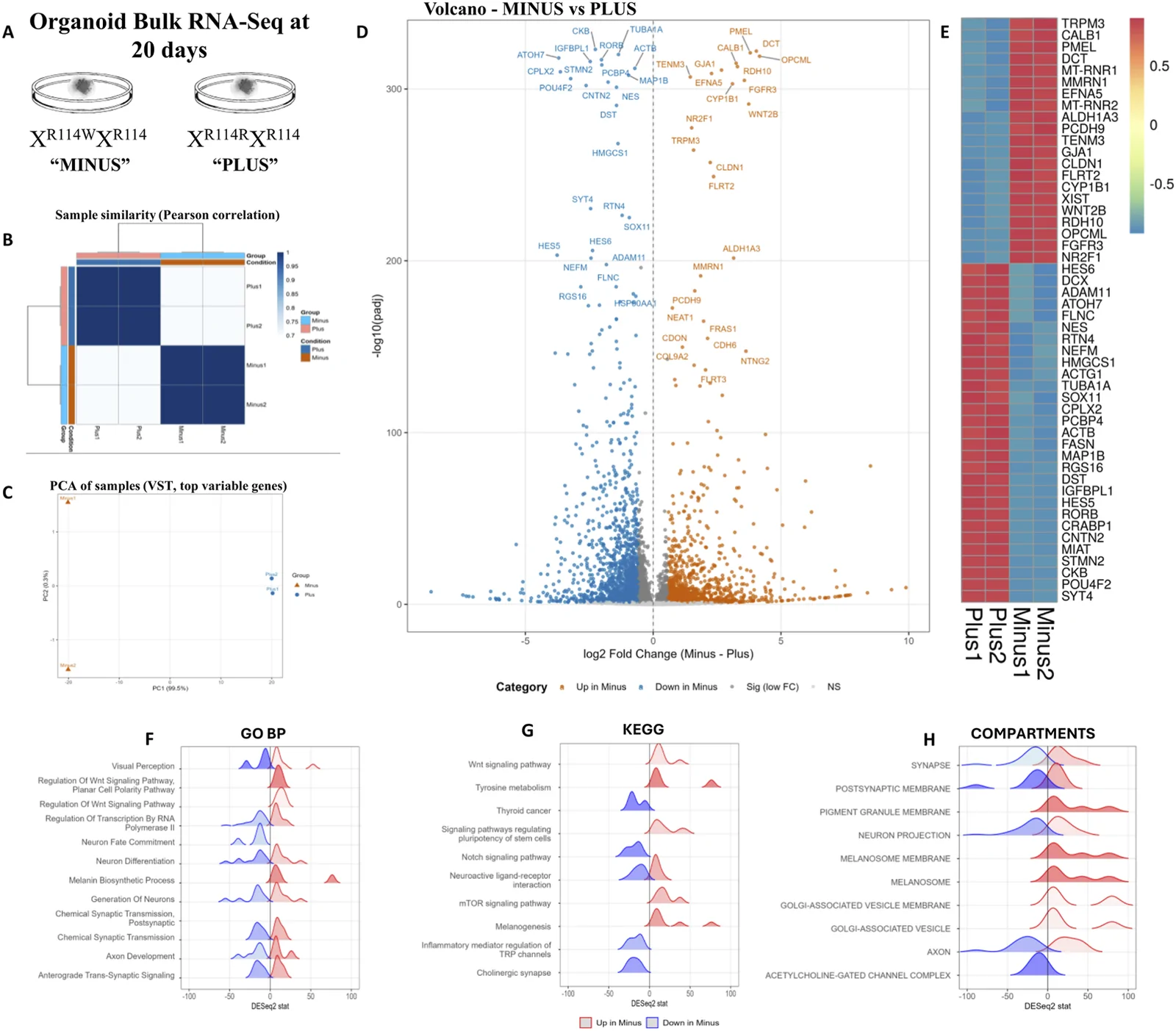
HNRNPH2-R114W organoids display a genotype-specific transcriptomic shift as determined by bulk RNA-Seq. **(A)** Schematic of the study design, indicating bulk RNA-seq profiling of Minus and Plus organoids on day 20. **(B)** Sample-to-sample similarity is evaluated using a Pearson correlation heatmap computed on a variance-stabilized (VST) expression matrix of the top 500 most variable genes, demonstrating within-genotype concordance and between-genotype separation. **(C)** The global sample structure is visualized using principal component analysis (PCA) based on the same VST matrix, highlighting genotype-associated separation. **(D)** Differential expression is depicted in a volcano plot for Minus versus Plus organoids using DESeq2, with effect size (log2 fold change) and significance (BH-adjusted p-value); significant genes are defined by padj <0.05 and baseMean ≥10, with log2 fold-change shrinkage applied for visualization where indicated. **(E)** Heatmap displays the top differentially expressed genes across all samples on the VST scale. **(F–H)** Functional enrichment analyses for genotype-associated expression changes are presented as ridge or peak-density panels for GO Biological Process, KEGG pathways, and subcellular compartment annotations, respectively. Gene-level preprocessing includes removing genes with fewer than 10 total reads and collapsing duplicate gene identifiers after Ensembl version stripping. Bulk RNA-seq analyses were performed using one pooled organoid RNA sample per genotype, with each pool generated from approximately 12 cerebral organoids collected 20 days after maturation and sequenced in two technical reactions per genotype. Differential gene expression was assessed using DESeq2. Genes were deemed significant if the Benjamini-Hochberg (BH)-corrected p-value was below 0.05 and the baseMean was at least 10. Plotting thresholds are specified where applicable.

The effect size and statistical significance are shown in a volcano plot (Figure 2D; Supplementary Figure S3E), which covers a large number of differentially expressed genes and displays approximately equal numbers of upregulated and downregulated transcripts at an FDR of 0.05 and a |log2FC| of at least 0.58. A heatmap of the top 50 differentially expressed genes also revealed distinct signature patterns (Figure 2E; Supplementary Figure S3D), among these genes that are involved in both neurodevelopment and pigment pathways. The top genes, for example *CNTN2, TUBA1A, CRABP1*, and *RDH10*, were found to be major targets of differential splicing, indicating an overlap between changes in gene expression and splicing (Figure 3).

**FIGURE 3:**
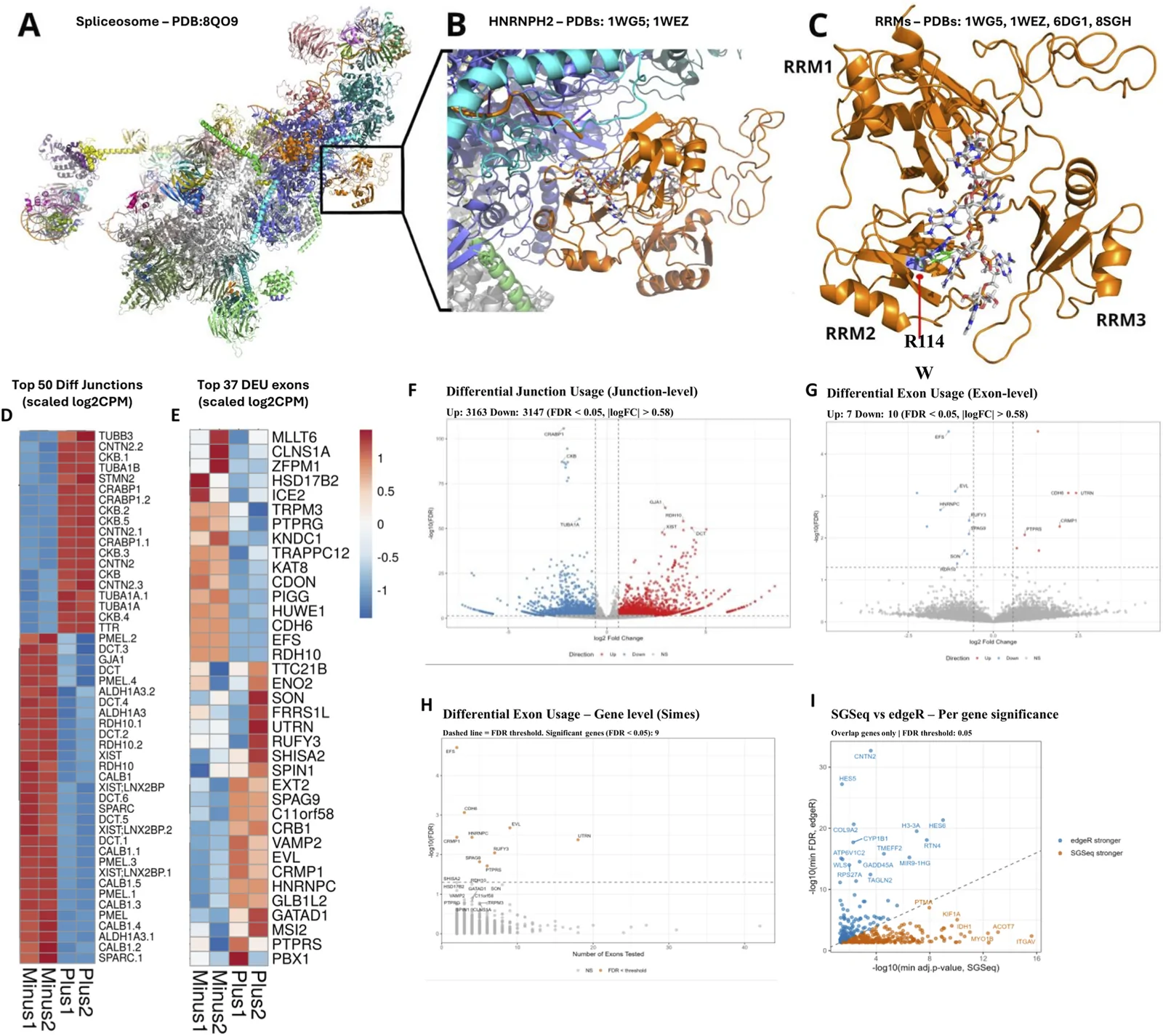
Splicing dysregulation in HNRNPH2-R114W organoids is predominantly junction-driven and only partially detected by exon-centric analyses. **(A)** Computational structural docking model depicting HNRNPH2 within the spliceosomal context (spliceosome PDB 8QO9), shown as an orange cartoon with the docking region boxed. **(B)** Enlarged view of the boxed region from **(A)** illustrates the inferred HNRNPH2–spliceosome interface and the predicted continuity of the modeled HNRNPH2-associated RNA path into spliceosomal RNA. **(C)** Computational structural model assembled from PDB entries 1WG5, 1WEZ, 2LEB, 4C0O, and 6HPJ highlights residue 114 near the predicted RNA-binding cleft (R114 highlighted; W114 mutation overlaid), illustrating a computationally inferred RNA-contact interface for the R114W substitution. **(D)** Heatmap displays the top 50 differentially used splice junctions across Minus and Plus samples (logCPM/log2CPM scale with prior count; row-scaled for visualization). **(E)** The heatmap shows the top differentially used exons across samples on the same scale. **(F)** Genome-wide junction-centric differential usage results are derived from junction discovery and quantification, including annotated and novel junctions supported by split reads, and tested with limma-trend and empirical Bayes moderation; significance is defined at BH FDR <0.05, with direction labels optionally using |log2FC| ≥ 0.58 for plotting. **(G)** Exon-centric differential usage results are based on exon counts generated with featureCounts using strand-specific counting and multimappers excluded, normalized by TMM, filtered to retain exons with at least five counts in at least two samples, and tested using voom and diffSplice, where significance is defined at BH FDR <0.05. **(H)** Gene-level aggregation of exon-centric splicing signals is performed using a Simes framework to summarize differential exon usage by gene. **(I)** The concordance overview compares gene-level splicing signals across junction-centric and exon/feature-centric workflows, highlighting both overlaps and method-specific detections.

Functional enrichment analysis using GO, Reactome, Compartments, KEGG, and Hallmark annotations (Figures 2F–H; Supplementary Figures S3G–I) indicated that genes downregulated in Minus organoids were associated with neurodevelopmental processes, including neuron differentiation, axon development, and synaptic transmission. Conversely, genes upregulated in Minus were associated with WNT/NOTCH signaling and pigment pathways, including melanogenesis, tyrosine metabolism, and retinoid biosynthesis. These results demonstrate broad genotype-associated changes in gene expression across neurodevelopmental pathways and provide a foundation for further investigation of differences in alternative splicing between groups.

### 2.4 Widespread alternative splicing rewiring in HNRNPH2-R114W organoids

A computational structural model related to the HNRNPH2-R114W splicing phenotype is provided in Figures 3A–C. In this model, HNRNPH2 is positioned relative to a spliceosomal assembly (spliceosomal B complex; PDB 8QO9) to illustrate a plausible spatial relationship between HNRNPH2 RNA engagement and the spliceosomal RNA framework. The HNRNPH2 RNA-binding module is represented by a composite RRM1-RRM3 structure derived from available HNRNPH2 templates listed in the figure legend (PDBs: 1WG5, 1WEZ, 6DG1, 8SGH). At the local interface level, the model highlights the RRM2 component residue R114 near the predicted RNA-recognition surface within the RRM2-RRM3 region. Based on short MD refinement and structural superposition with related RNA-recognition protein templates (SRSF1 and SRSF2; PDBs: 6HPJ, 4C0O, 2LEB), R114 was positioned in a geometry compatible with potential RNA-contact interactions, whereas the R114W substitution was predicted to alter this interface. The precise RNA hairpin or duplex configuration and the global spliceosome docking remain computational predictions that require extended simulations and direct structural validation through methods such as cryo-electron microscopy. Thus, docking analyses informed by available HNRNPH2 RRM1-3 structures and RNA-recognition protein templates provide a hypothesis-generating structural framework for interpreting how the HNRNPH2-R114W mutation at the RRM2 interface may alter RNA recognition during splicing (Figures 3A–C).

The effect of the HNRNPH2-R114W pathogenic variant on alternative splicing was assessed by means of two complementary BAM-based splicing analysis workflows that were based on the same bulk RNA-seq alignments. In the first approach, a junction-focused workflow employed SGSeq to identify and quantify both known and newly detected splice junctions supported by split reads, after which differential junction usage was tested using limma-trend and empirical Bayes moderation. In the second approach, an exon-focused workflow used featureCounts to quantify the annotated exon-level features, followed by voom/limma diffSplice testing and Simes-based gene-level aggregation. These two workflows thus constitute complementary analyses of the same RNA-seq dataset, the junction-focused analysis records local donor-acceptor usage, while the exon-focused analysis examines annotated exon usage and the gene-level exon-usage burden. The results were presented in the form of junction-, exon-, and gene-level significance plots, as well as heatmaps showing the scaled log2 CPM values for the most significantly differentially used junctions and exons (Figures 3D–I; Supplementary Figure S4).

Heatmaps of the top 50 differential junctions and top 37 differentially used exons revealed distinct genotype-specific clustering (Figures 3D,E). Plus samples exhibited increased junction usage in *TUBA1A, TUBB3, CNTN2*, and *CKB*, while Minus samples showed higher junction usage in *DCT, PMEL, ALDH1A3, RDH10, CALB1*, and *XIST*. This separation indicates a strong splicing pattern associated with the HNRNPH2-R114W genotype. Minus-biased exon usage was enriched for genes involved in calcium and developmental signaling, including *TRPM3, KNDC1, CDH6, EFS*, and the retinoic acid pathway enzyme *RDH10*. In contrast, Plus-biased exon usage was enriched for genes related to neuronal vesicle trafficking (*VAMP2*), cytoskeletal remodeling and actin dynamics (*EVL, CRMP1*), and RNA or chromatin regulation (*HNRNPC, MSI2, GATAD1*).

The junction-level analysis found more than 6,000 junctions that were significantly altered (with an FDR less than 0.05 and a |log2FC| greater than or equal to 0.58) out of the approximately 98,000 that were examined, corresponding to about 6%–7% of all junctions, as shown in the junction-level volcano plot (Figure 3F), this plot displaying widespread and balanced changes in splicing, with thousands of junctions being regulated in both directions. On the other hand, the exon-level analysis revealed only 17 significant exons (Figure 3H; Supplementary Figures S4D–F). This pattern shows that the splicing signal associated with the genotype was mainly detected at the splice-junction level, whereas the exon-level aggregation identified only a smaller number of events. The fact that junctions dominate in this way is in agreement with the known role of HNRNP H-family proteins in binding to G-rich RNA and in the regulation of splice sites, and also probably reflects the higher local resolution achieved by junction-focused analyses as compared to exon-focused ones (Caputi and Zahler, 2001; Huelga et al., 2012; Pervouchine et al., 2013; Goldstein et al., 2016; Brownmiller and Caplen, 2023). Using a Simes-type framework for gene-level analysis of exon usage, nine genes were identified that had significant changes at the exon level, namely *CDH6, EFS, EVL, HNRNPC, UTRN, CRMP1, RUFY3, SPAG9*, and *PTPRS*, all of which encode proteins involved in neuronal projection, axon development, extracellular matrix organization, and cytoskeletal remodeling (Figure 3H).

To differentiate genes with only differential expression (DE), only differential exon usage (DEU), or both, results were categorized as follows: 1,966 genes were DE only, seven genes were DEU only, and two genes exhibited both differential expression and exon usage alterations (Supplementary Figures S4A–C). The seven DEU-only genes, *RUFY3, EFS, SPAG9, CRMP1, HNRNPC, UTRN*, and *EVL*, displayed significant splicing changes without detectable expression shifts, indicating regulation primarily at the post-transcriptional level. *CDH6* and *PTPRS* were identified as genes with combined transcriptional and exon-usage alterations (Supplementary Figure S4B).

The degree of agreement between junction-focused and exon-focused workflows was assessed by comparing the per-gene significance for genes that were significant in both methods (Figure 3I; Supplementary Figures S4D–F). Genes including *CNTN2, HES5, CYP1B1, COL9A2, IFT172, CLASP1*, and *TMEFF2* showed consistent significance in both analytical approaches. The agreement between the two methods supports the analytical consistency of the genotype-associated splicing differences. It indicates that the main signal is not restricted to a single representation of splicing, since it appears in both the junction-focused and exon-focused workflows.

Genome-wide SGSeq analysis of differential usage at the feature level confirmed and extended junction-based findings by identifying significant shifts in feature usage (see Supplementary Figure S4D). In this context, a feature represents a local splice-graph element derived from observed exon–junction structures, enabling detection of complex usage changes not captured by single annotated junctions. Significant features were identified in genes such as *VIPR1, BSDC1, ITGAV, SNX17, SELENON, GAD1*, and *KCNN3*, which are associated with vesicle membrane function, actin-based cell projection, and transmembrane transport of inorganic cations. These results align with coordinated alterations in membrane trafficking, neurite-associated structural organization, and ion-handling pathways that exhibit genotype-related differences in usage. Collectively, the analyses demonstrate extensive splicing remodeling at the junction level in HNRNPH2-R114W variant organoids, with a more limited number of changes at the exon level, providing a multi-layered perspective on splicing heterogeneity.

### 2.5 Integrated RNA-layer metrics identify priority candidates and functional convergence

In order to organize the results obtained at both the transcriptional and RNA-processing levels, we developed an integrated, gene-focused prioritization framework that combined evidence from differential expression (DE), event-level (that is, junction-level), and isoform-level analysis (Figure 4; Supplementary Figure S5). The event-level evidence shows differences in the use of individual splice junctions, while the isoform-level evidence indicates changes in the relative abundance of the full-length transcript isoforms. For each gene, the RNA-processing signals were merged with transcriptional significance to produce a comprehensive prioritization score that summarizes the overall activity at the RNA level.

**FIGURE 4:**
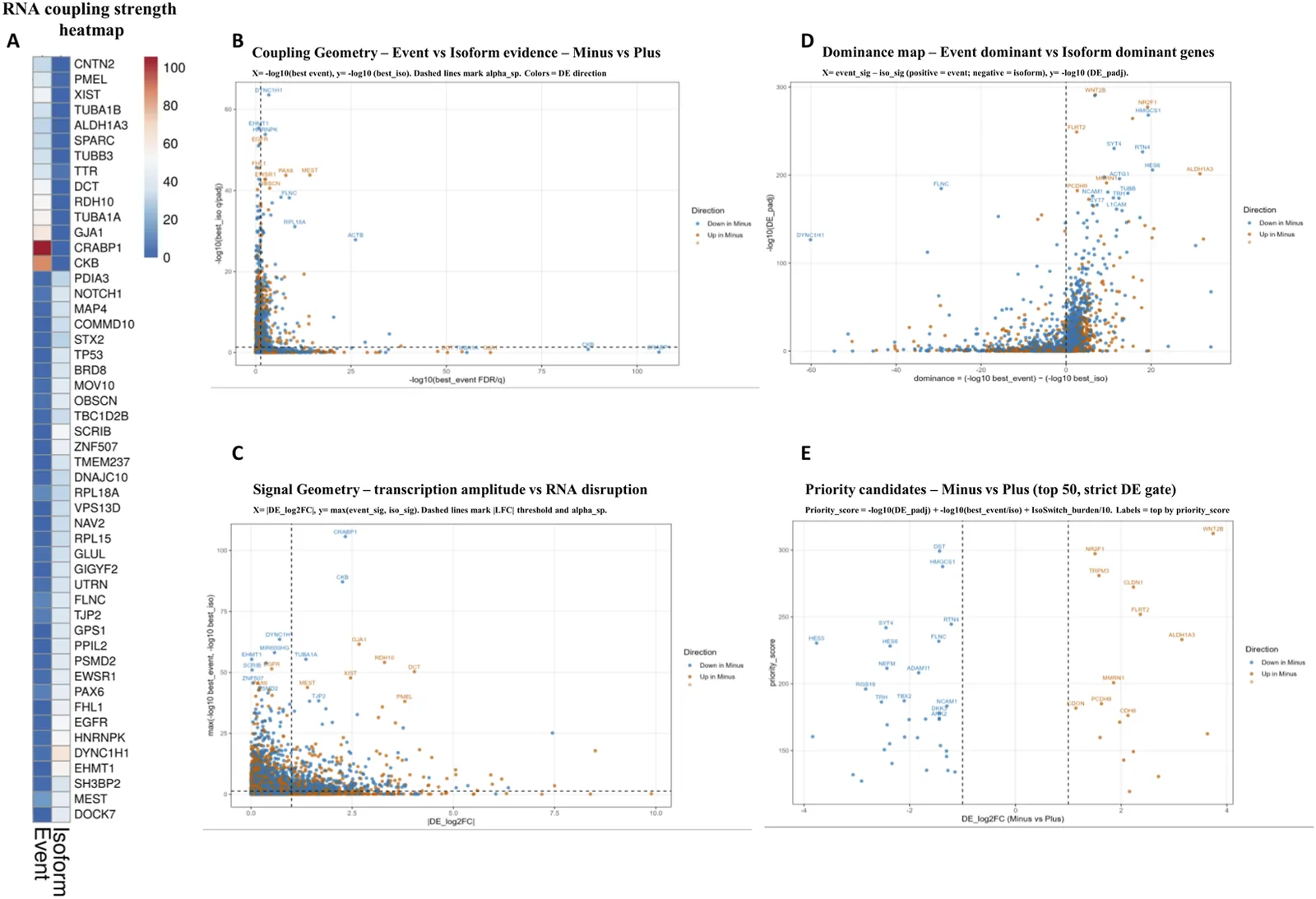
Integrated RNA-layer prioritization connects differential expression with splicing and isoform remodeling, revealing convergent functional programs. **(A)** The coupling-strength heatmap summarizes integrated multi-layer RNA remodeling signals for prioritized genes, combining gene-level differential expression with event- and isoform-level evidence. **(B)** Coupling-geometry scatterplot positions genes by event-level versus isoform-level remodeling, distinguishing those dominated by junction or exon events from those dominated by isoform switching. **(C)** Signal-geometry scatterplot contrasts transcriptional amplitude with RNA disruption, separating expression-dominant from splicing or isoform-dominant effects. Prioritization employs harmonized Ensembl gene identifiers, version-stripped with duplicates collapsed, and applies a uniform integration threshold, where DE is defined as padj <0.05, baseMean ≥10, total read count ≥10, and |log2FC| ≥ 1.0, with splicing or isoform significance assessed at FDR <0.05. Event-level inputs include exon differential usage (diffSplice) and junction differential usage by SGSeq-derived junction usage testing, while isoform-level inputs are aggregated from isoform-focused frameworks implemented in the pipeline. **(D)** Dominance map contrasts event-level versus isoform-level evidence, separating event-dominant from isoform-dominant genes in Minus versus Plus organoids. **(E)** Priority-candidate plot ranks integrated RNA-layer candidates by combining differential expression, event-level, and isoform-level evidence under the strict differential-expression gate.

The heatmap of RNA coupling strength summarizes, with respect to the top-ranked genes, the relative sizes of the event-level and isoform-level RNA signals (Figure 4A). It shows the varying degrees of contribution from the different layers among the prioritized genes, identifying those that have event-dominant, isoform-dominant, and mixed coupling patterns, such as *CNTN2, PMEL, XIST, TUBA1A, CRABP1*, and *CKB*.

The coupling geometry used to examine the relationship between event-level and isoform-level evidence is shown in Figure 4B; in this arrangement genes are plotted with their highest event-level significance on the x-axis and their highest isoform-level significance on the y-axis. This type of representation divides the prioritized genes into groups showing support at both levels and those mainly driven by one layer, the points moving towards one axis when the relevant signal is dominant. The genes marked in the plot serve to illustrate these coupling regimes, namely *DYNC1H1, EHMT1, HNRNPK, ACTB, CRABP1* and *CKB*.

To connect RNA-processing disruption with transcriptional amplitude, a signal geometry analysis plots the absolute value of the DE effect size against an RNA-disruption metric which is defined as the maximum of the event- or isoform-level significance for each gene (Figure 4C). The plot shows a distinction between genes that exhibit large changes in expression but only moderate RNA-processing signals and genes that have relatively small changes in expression together with a strong RNA-level perturbation, with representative loci demonstrating these categories such as *CRABP1, CKB, TUBA1A, GJA1, RDH10,* and *PMEL*.

The dominance map shows the relative strength of evidence at the event level compared to that at the isoform level by plotting the numerical difference as a dominance score against the overall significance of differential expression (Figure 4D). If the dominance value is positive, this means that the gene is event-dominant; a negative value indicates that the gene is isoform-dominant, and values that are intermediate show a gradual transition between these two extremes. Examples of event-dominant genes are *HES6, RTN4* and *SYT4*, while *FLNC* and *DYNC1H1* show isoform-dominant patterns and *NCAM1, L1CAM* and *TRH* are found in intermediate positions.

The candidates were identified by plotting the transcriptional effect sizes against the composite prioritization score (Figure 4E), with the most highly ranked genes being highlighted under a strict DE gate, this gate being defined by a significant DE-adjusted p-value and an absolute log2 fold-change threshold. Among the top-ranked genes that were highlighted under this gate are *TRPM3, WNT2B, NR2F1, CLDN1, FLRT2, ALDH1A3, HMGCS1, DST,* and *RTN4*.

The functional enrichment analysis of the gene sets that were given priority was carried out separately for GO Biological Process, Molecular Function, and Cellular Component (see Supplementary Figures S5B–D). In the case of Biological Process, the most prominent signals are those relating to neuronal differentiation and projection programs, such as axon guidance and neuron projection morphogenesis, together with translation and peptide biosynthetic processes, and the various structural programs involving extracellular matrix and cilia organization. With respect to Molecular Function, the enriched terms highlight cytoskeletal, adhesion-associated, and RNA-binding activities. As for Cellular Component, the enriched categories are pigment granule and melanosome compartments, as well as the nucleus, focal adhesion, and the collagen-containing extracellular matrix.

### 2.6 Cell-type composition shifts in HNRNPH2-R114W organoids

To assess overall changes in cell-type composition in HNRNPH2-R114W organoids, bulk RNA-seq samples were analyzed using the MuSiC deconvolution method in combination with the HNOCA human neural organoid atlas, enabling estimation of cell-type proportions for each sample (Wang et al., 2019; He et al., 2024). Both Minus and Plus organoids contained a mixture of neuroepithelium, dorsal and ventral telencephalic neuronal populations, glioblasts, oligodendrocyte progenitor cells (OPCs), astrocytes, and markers for non-telencephalic neuron composition (Figures 5A–C). Genotype-associated compositional differences indicated that neuroepithelium and glial-rich populations (glioblasts, OPCs) were enriched in Minus organoids, as reflected by negative delta fractions for the CRISPR − variant. In contrast, certain neuronal populations exhibited positive delta values, indicating a slight reduction in neuronal numbers in Minus organoids. On average, the neuronal fraction in Minus organoids was approximately 5% lower than in Plus organoids (Figure 5B).

**FIGURE 5:**
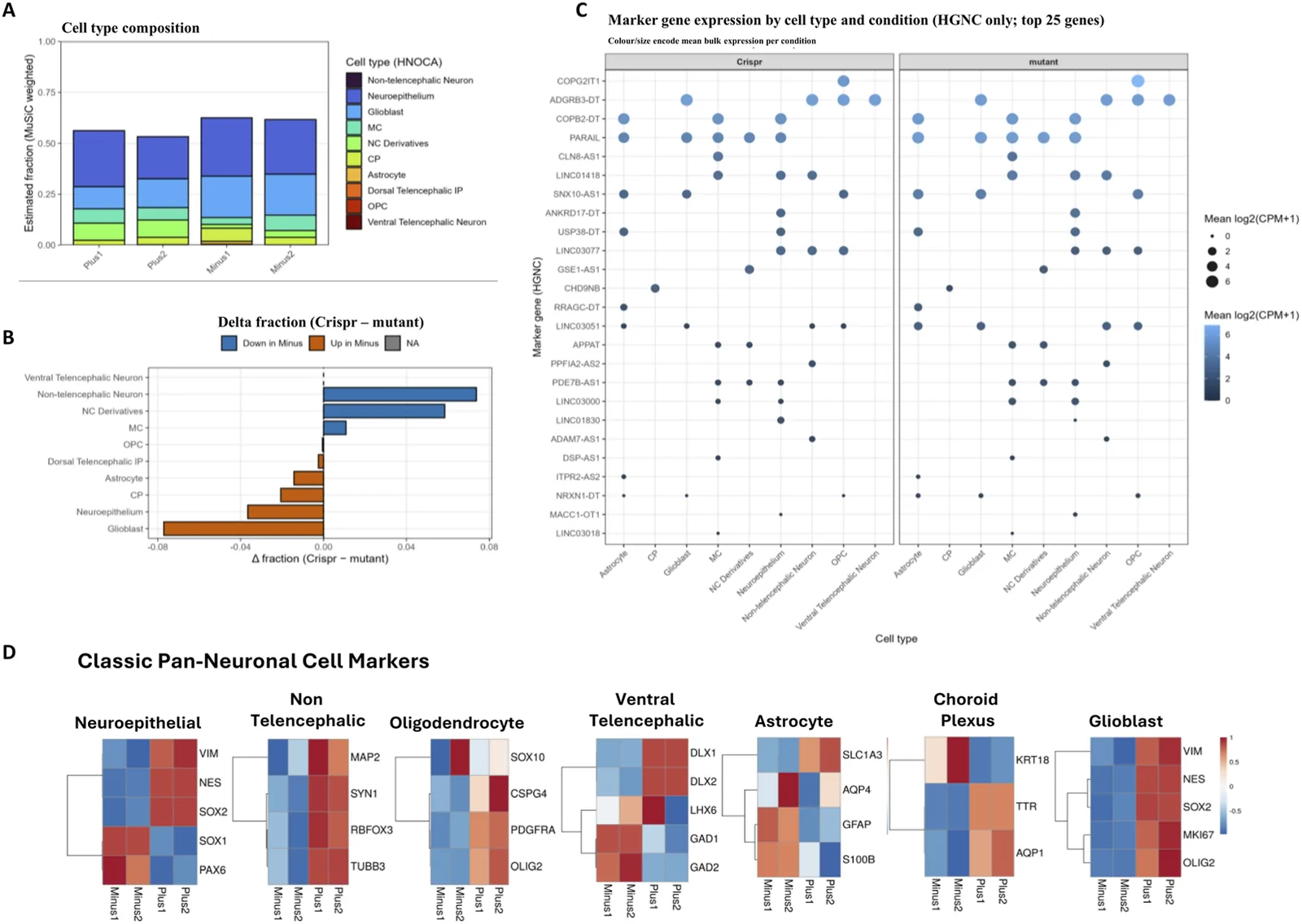
MuSiC deconvolution and marker-program analysis support modest inferred differences in cell-type composition. **(A)** Stacked cell-type fraction plot showing MuSiC-inferred composition per sample for Minus and Plus organoids, estimated by weighted nonnegative least squares regression against a neurodevelopmental single-cell reference (HNOCA) using a shared gene domain (bulk ∩ reference; optionally intersected with MuSiC “used genes”), and retaining cell-type groups supported by a minimum number of 5 cells per donor. **(B)** Delta-fraction summary of CRISPR-corrected (plus) versus HNRNPH2 R114W (minus) showing genotype-associated composition shifts across inferred cell types. MuSiC is run on raw (untransformed) gene-level counts, with Ensembl version stripping and duplicate collapsing. **(C)** Marker‐gene expression dot plots comparing CRISPR‐corrected and mutant conditions across inferred cell types, with mean log2(CPM+1) expression encoded by point size and color. **(D)** Clustered heatmaps showing conventional marker‐gene expression across seven inferred cell‐type groups; values are based on log2(CPM+1), centered within each sample, and z‐scaled for visualization. Cell-type fractions were inferred from bulk RNA-seq using MuSiC deconvolution against the HNOCA human neural organoid reference atlas. Results represent deconvolution-based estimates from pooled RNA-seq samples with technical sequencing replication, not direct single-cell measurements.

The high level of genotype-specific correlations for the MuSiC-inferred fractions showed that the compositional estimates were stable across the genotype-specific sequencing reactions, whereas the lower level of correlations between genotypes suggested differences in the inferred cell-type composition that were associated with genotype (Supplementary Figure S6). The pattern of these correlations indicates that the inferred compositional changes are consistent across the technical sequencing reactions.

To further assess whether inferred compositional differences were reflected at the expression level, we examined curated marker gene heatmaps stratified by major lineage classes (Figure 5D). Neuroepithelial and progenitor-associated markers, including *PAX6* and *SOX2*, astrocyte markers *GFAP, S100B*, and *AQP4*, displayed genotype-linked expression patterns consistent with the MuSiC-inferred enrichment of progenitor or glial populations in Minus organoids. Conversely, glioblast markers including *MKI67, OLIG2*, and the OPC markers *CSPG4* and *PDGFRA* showed reduced expression in Minus samples, in agreement with the modest reduction in neuronal fractions inferred by deconvolution.

Collectively, the convergence of MuSiC-based cell-type fraction estimates, lineage-specific marker heatmaps, and expression changes in canonical cell-type markers supports an association between HNRNPH2-R114W dysfunction and modest inferred compositional biases favoring neuroepithelial and glioblast lineages over specified neuronal populations. Genotype-linked alterations in canonical lineage marker expression are consistent with these inferred compositional shifts. Therefore, while cell-type composition should be considered when interpreting bulk transcriptomic and splicing data, the relatively small magnitude of compositional differences, approximately a 5% reduction in neuronal specification, suggests that the major gene expression and splicing changes observed in HNRNPH2-R114W organoids are unlikely to be explained solely by alterations in cellular composition.

## 3 Discussion

The early stages of corticogenesis are critical for investigating neural development and for developing therapeutic strategies targeting mutations in the HNRNPH2 gene. In this study, isogenic cerebral organoids derived from a patient-specific induced pluripotent stem cell (iPSC) line carrying the rare HNRNPH2-R114W variant (Minus) and its CRISPR-corrected counterpart (Plus) serve as a robust system to examine RNA-mediated disease mechanisms under controlled genetic conditions. Both genotypes retain pluripotency and generate comparable numbers of PAX6-positive progenitors and TUBB3-positive neurons (Figures 1B–D; Supplementary Figure S2), demonstrating that early corticogenesis is preserved *in vitro*. The comparable intensity of PAX6 and TUBB3 signals, together with modest differences in TYR, G3BP1, and SHANK1 levels between genotypes, suggests that the HNRNPH2-R114W variant selectively influences stress, pigment, and synaptic marker profiles without substantially affecting early neuronal output (Supplementary Figures S2J–L). Although immunofluorescence targeting HNRNPH2 revealed genotype-dependent differences in staining distribution, this assay does not determine whether the R114W variant alters HNRNPH2 mRNA stability, translation efficiency, or total protein abundance. Thus, the HNRNPH2 staining pattern serves as an indirect indicator of genotype-associated differences in HNRNPH2 distribution, rather than providing a direct measure of HNRNPH2 dosage. Given the maintained populations of PAX6-positive progenitors and TUBB3-positive neurons, these findings support the use of these organoids as a stable model for investigating downstream effects of rare variants through RNA-mediated mechanisms and underscore the utility of organoid models in recapitulating early neural development within isogenic backgrounds relevant to neurodevelopmental disorders.

Mutations in *HNRNPH2* result in an X-linked neurodevelopmental disorder characterized by developmental delay, intellectual disability, Rett- or autism-like features, epilepsy, and variable structural brain abnormalities. These disorders frequently arise from missense mutations in the PY-NLS and qRRM domains (Bain et al., 2016; Pilch et al., 2018; Gillentine et al., 2021). Comprehensive analyses of the HNRNP family indicate that *HNRNPH2* variants produce overlapping neurodevelopmental phenotypes, particularly those involving autism spectrum disorder features, by disrupting convergent molecular pathways (Gillentine et al., 2021; Brownmiller and Caplen, 2023). HNRNP H/H2 proteins specifically bind G-rich RNA sequence motifs, thereby regulating splicing, 3′-end processing, and ribonucleoprotein complex assembly (Huelga et al., 2012; Honoré et al., 1995; Caputi and Zahler, 2001; Brownmiller and Caplen, 2023). Studies employing patient and neuronal models suggest that toxic gain-of-function effects and protein mislocalization underlie the disruption of neural splicing (Brownmiller and Caplen, 2023; Larizza et al., 2022). Recent organoid studies extend these mechanistic models to human cortical tissue and support the interpretation of layered RNA signatures in the context of RNA-binding protein dysfunction.

The distinct clustering of bulk transcriptomes by genotype (Figure 2C; Supplementary Figure S3) demonstrates strong transcriptional separation between HNRNPH2-R114W and CRISPR-corrected organoids within this isogenic model. Although principal component 1 (PC1) accounted for a substantial proportion of total variance, this primary axis was consistent with results from differential expression, pathway enrichment, and splicing analyses, supporting a coherent genotype-associated transcriptomic profile. The greater enrichment of neuronal and synaptic components compared to pigment and retinoid programs (Figures 2D–H; Supplementary Figure S3) indicates that the HNRNPH2-R114W mutation is associated with organized transcriptomic reprogramming in cortical organoids, in agreement with previous studies of HNRNPH2 and related HNRNP disorders (Raj and Blencowe, 2015; Weyn-Vanhentenryck et al., 2018; Irimia et al., 2014; Gillentine et al., 2021; Brownmiller and Caplen, 2023; Larizza et al., 2022). Given that the transcriptomic analyses were derived from technical sequencing replicates obtained at a single developmental time point from a pooled organoid RNA sample per genotype, these bulk RNA-seq findings are interpreted as exploratory while providing a focused molecular framework for examining how the observed signatures evolve across developmental time and recur across independently generated biological samples.

Splicing analysis reveals that the HNRNPH2-R114W mutation primarily affects RNA processing at the splice-junction resolution stage. These alterations are only partially detected by exon-based tests and are largely independent of overall gene expression changes (Figures 3D–G). Junction-level changes are more prevalent than exon-level changes, likely reflecting HNRNPH2’s function as a splice site regulator and the increased sensitivity of junction-focused methods, rather than widespread exon loss. Given that HNRNP H family members bind G-rich RNA elements and participate in splice-site selection, a junction-focused pattern is biologically plausible. Junction-focused methods directly assess local donor-acceptor usage, whereas exon-focused tests aggregate broader exon-level read counts and may identify fewer events (Pervouchine et al., 2013; Goldstein et al., 2016; Huelga et al., 2012; Caputi and Zahler, 2001; Brownmiller and Caplen, 2023). The relatively high number of affected junctions for a single mutation (R114W) in an RNA-binding protein (Figure 3F) suggests that local junction usage alterations can drive widespread isoform reconfiguration across the transcriptome. Unsupervised heatmaps of junction and exon usage corroborate this, demonstrating strong genotype-based clustering, particularly for junction-related features (Figures 3D,E). These results are consistent with previous reports that mutations in HNRNP H-family regulators disrupt splicing networks involved in neural development, signaling, and metabolism (Raj and Blencowe, 2015; Ule and Blencowe, 2019; Huelga et al., 2012; Caputi and Zahler, 2001). The observed distinction between junction-level perturbations and gene-level abundance changes (Figures 3D–H) indicates that RNA processing defects can occur independently of changes in total gene expression, in line with findings from other models of HNRNPH2 and RNA-binding protein dysfunction (Gillentine et al., 2021; Brownmiller and Caplen, 2023; Larizza et al., 2022).

An integrated analysis of differential expression and differential exon usage (DE-DEU) elucidates the relationship between gene expression and splicing by demonstrating that only a limited subset of genes exhibits changes in exon usage without corresponding transcriptional alterations (Supplementary Figure S4). Notably, genes such as *RUFY3, EFS, SPAG9, CRMP1, HNRNPC, UTRN*, and *EVL* display a selective DEU-only pattern, consistent with post-transcriptional regulation of neuronal wiring and cytoskeletal pathways, and align with HNRNP-driven splicing networks and ASD-associated splicing regulators (Raj and Blencowe, 2015; Irimia et al., 2014; Brownmiller and Caplen, 2023). While most transcriptional changes do not result in pronounced exon-level remodeling, a distinct subset of transcripts exhibits either isolated or combined disruptions, providing insight into the mechanisms by which HNRNPH2-R114W connects gene expression changes with splicing defects.

Splicing event analysis using SGSeq, in conjunction with junction-focused tests, demonstrates that principal splicing signals are consistently detected across analytical methods, underscoring the extensive RNA rewiring dependent on HNRNPH2 (Figure 3I; Supplementary Figure S4). The junction- and exon-focused workflows identify overlapping primary targets, alongside method-specific findings, indicating that cooperative regulation by HNRNPs influences multiple aspects of RNA architecture, with each method capturing complementary features of the underlying spliceopathy (Ule and Blencowe, 2019; Huelga et al., 2012; Robinson et al., 2010; Goldstein et al., 2016). This concordance supports the interpretation that the HNRNPH2-R114W mutation is associated with widespread yet organized splicing disruptions during cortical tissue development, rather than isolated, method-specific artifacts.

Despite limited convergence, the integrated RNA-layer framework provides structured and informative insights into disease mechanisms (Figures 4A–D). This approach combines data on gene expression, splicing, and isoform switching to identify priority candidates implicated in both transcriptional and post-transcriptional dysregulation (Figure 4E). For instance, genes such as *CRABP1, CKB, TUBA1A, CNTN2, NCAM1*, and *TRPM3* exhibit multi-layer signatures, whereas others are predominantly affected at the isoform or junction level (Figures 4B–E; Supplementary Figure S5). These results support the concept that RNA-binding proteins influence both global isoform processing and localized RNA splicing events (Caputi and Zahler, 2001).

Key genes such as *CNTN2* and *NCAM1*, which are critical for cortical wiring, display multi-layer perturbations and are linked to axon guidance and cell adhesion in cortical development and autism spectrum disorder. Additional genes, including *TRPM3, HES5, HES6, RTN4*, and *HMGCS1*, are involved in neuronal excitability, neurogenesis, and metabolic support. Enrichment analyses identify pathways related to axon guidance, morphogenesis, and synaptic function (Supplementary Figures S5B–D). These results nominate a select group of multi-layer genes as candidate hubs of HNRNPH2-R114W-dependent dysregulation, offering potential endpoints for future mechanistic studies and for assessing RNA-targeted interventions such as antisense oligonucleotides.

MuSiC deconvolution and pan-neuronal marker analyses (Wang et al., 2019; He et al., 2024) reveal subtle shifts in inferred cell-type proportions rather than extensive lineage turnover (Figures 5A–D). Minus organoids exhibit increased neuroepithelial and glial-associated fractions and decreased neuronal fractions, with curated marker heatmaps supporting the biological plausibility of these changes (Figure 5D). These compositional differences suggest that HNRNPH2-R114W may bias developmental trajectories toward progenitor or glial-associated states and away from neuronal specification, consistent with delayed or altered neurogenic timing rather than neuronal loss. This interpretation is consistent with clinical data indicating microcircuit dysfunction and ASD/ID, but not microcephaly (Bain et al., 2016; Pilch et al., 2018; Gillentine et al., 2021; Brownmiller and Caplen, 2023).

Although these inferred changes in cell composition may partially explain lineage-associated differences in gene expression, their modest magnitude relative to the overall gene expression and splice junction alterations suggests that cell composition alone is unlikely to account for the entire molecular phenotype. As MuSiC estimates cell-type proportions indirectly from bulk RNA-seq using a single-cell reference map, these results should be interpreted as supporting inferences about cell composition rather than providing direct single-cell measurements. Collectively, both the deconvolution and marker gene analyses support a model in which the HNRNPH2-R114W mutation is associated with only a minor bias in neurodevelopmental cell composition, alongside more extensive genotype-related dysregulation of transcription and splicing.

In summary, this study demonstrates that the HNRNPH2-R114W rare-variant brain organoid phenotype is characterized by preserved early neuronal differentiation, broad yet organized expression changes, junction-dominant splicing rewiring, and convergence on key gene hubs, with cell-composition shifts functioning as modifiers rather than primary drivers of differentiation. These findings support models in which *HNRNPH2* variants disrupt post-transcriptional regulation through altered protein localization, splicing, and ribonucleoprotein assembly, resulting in toxic or dominant-negative effects rather than simple loss of function (Bain et al., 2016; Gillentine et al., 2021; Brownmiller and Caplen, 2023). PY-NLS and qRRM mutations may impair nuclear import and RNA binding, thereby limiting HNRNPH1 compensation and inducing toxic gain-of-function effects in neural splicing networks (Gillentine et al., 2021; Brownmiller and Caplen, 2023). The organoid model provides experimental support for this perspective and may serve as a platform for evaluating allele-selective antisense oligonucleotide strategies, *HNRNPH2* modulation, or approaches to assess paralog compensation. This integrative methodology, which combines differential expression, multi-layer splicing analysis, and single-cell-based bulk RNA-seq deconvolution, is likely applicable to other RBP-driven neurodevelopmental disorders.

## 4 Methods

### 4.1 Generation and differentiation of human iPSC lines, CRISPR correction, and cerebral organoid culture

Peripheral blood mononuclear cells (PBMCs) were obtained from an individual harboring a pathogenic *HNRNPH2* rare variant and reprogrammed into induced pluripotent stem cells (iPSCs) using non-integrating episomal vectors under feeder-free conditions (Applied Stem Cell, Inc.). An isogenic control line was established by correcting the HNRNPH2-R114W locus via CRISPR-Cas9 within the same genetic background. Sanger sequencing at the DNA level revealed correction-associated sequence changes at the targeted *HNRNPH2*-R114W locus in CRISPR-edited positive clones compared with the parental line (Supplementary Figure S1). The *HNRNPH2* rare variant iPSC line served as the parental line for all subsequent stem cell and organoid differentiation experiments.

iPSCs were cultured in mTeSR medium on plates coated with growth factor-reduced Matrigel at 37 °C, 5% CO2, and greater than 90% relative humidity. Medium was replaced daily. Upon reaching 70%–80% confluence, cells were passaged using Accutase and reseeded at empirically determined densities to preserve pluripotent morphology. Temporary treatment with the ROCK inhibitor Y-27632 was applied after passaging, particularly following dissociation into single cells. Pluripotency of both the HNRNPH2-R114W variant and CRISPR-corrected iPSC lines was evaluated by colony morphology, growth stability, absence of extensive differentiation, and immunostaining for NANOG, SOX2, and OCT4.

To minimize variability associated with passage, organoid differentiation was initiated using iPSC cultures at passage 13. Prior to embryoid body formation, both rare variant-containing and CRISPR-corrected lines were matched for passage number and culture conditions. At this stage, colonies exhibited dense morphology, a high nuclear-to-cytoplasmic ratio, and minimal edge differentiation. Passage 13 was selected based on pilot studies demonstrating stable expression of pluripotency markers between passages 10 and 15.

Cerebral organoids were generated using a modified Lancaster protocol (Lancaster et al., 2013) in conjunction with the Stem Cell Technologies Brain Organoid Kit. The procedure included embryoid body formation, neural induction, embedding in Matrigel to facilitate neuroepithelial cell expansion, and maturation in suspension culture. For long-term growth, a CO2-resistant orbital shaker was employed in place of a spinning bioreactor.

### 4.2 Whole-mount immunofluorescence, confocal imaging, and image processing

Organoids were collected at developmental stages corresponding to early corticogenesis and cell network formation. Multiple organoids were harvested for each condition and time point. Samples were rinsed with PBS, fixed in 4% paraformaldehyde for 30–60 min at room temperature with gentle rocking, washed in PBS to remove fixative, and stored in PBS at 4 °C. For permeabilization, fixed organoids were treated with PBS containing 0.2%–0.5% Triton X-100 for 1–2 h at room temperature with gentle agitation. Blocking was performed in PBS containing 0.1%–0.2% Triton X-100, 5%–10% normal serum, and, when appropriate, 1%–3% BSA for 1–2 h at room temperature.

Organoids were stained *in situ* with primary antibodies targeting markers of cortical progenitors and neurons, including PAX6, SOX2, and TUBB3, as well as HNRNPH2 and additional study-specific proteins listed in the antibody table. Primary antibodies were diluted in blocking buffer at empirically determined concentrations, typically ranging from 1:200 to 1:1,000, and applied in sufficient volume to cover the organoids. Incubation was performed overnight at 4 °C with gentle rocking. Organoids were subsequently washed multiple times with PBS containing 0.1%–0.2% Triton X-100.

Secondary antibodies conjugated to fluorophores and compatible with the host species of the primary antibodies were diluted in blocking buffer and incubated at room temperature in the dark for 1–2 h. Organoids were washed repeatedly with PBS containing detergent. Nuclear counterstaining was performed using DAPI or Hoechst at standard concentrations for 10–20 min, followed by additional PBS washes. When necessary, organoids were incubated in a refractive-index-matching solution, such as PBS mixed with glycerol, prior to mounting.

Immunostained organoids were positioned in glass-bottom dishes or chamber slides with a minimal volume of PBS or mounting medium to prevent movement during imaging. Imaging was conducted using a Nikon Eclipse microscope equipped with ×2 and ×4 objectives and appropriate filter sets. Multiple fields or Z-positions were acquired per organoid to visualize relevant structures. Exposure and illumination settings were maintained consistently throughout each experiment.

Images were analyzed using NIS-Elements and FIJI/ImageJ. Only uniform brightness and contrast adjustments were applied per experiment. Maximum-intensity projections and tiled montages were generated as required. No non-linear, feature-selective, or region-specific modifications were performed. For certain single-channel immunofluorescence panels, grayscale images were converted to black and white and inverted so that the background appeared white and marker signal appeared black. This adjustment enhanced the visibility of marker patterns and tissue structures. Representative images displaying typical morphology and marker expression were selected, and original image data were preserved for quantitative analysis.

### 4.3 RNA-seq library preparation, alignment, differential expression, splicing, and RNA-layer integration

RNA-seq was performed using a single pooled RNA sample from organoids for each genotype. Each genotype-specific pool comprised approximately 12 cerebral organoids collected 20 days after maturation. The RNA input was normalized to ensure equal total RNA mass prior to library preparation. Each pooled sample underwent two technical sequencing reactions, resulting in two RNA-seq datasets per genotype for subsequent analysis. To isolate total RNA from each sample, 1.125 mL of TRIzol was added to 0.375 mL of sample. The 1.4 mL mixture was poured into a PhaseMaker tube, and RNA extraction was performed following the manufacturer’s protocol. Cleanup was done with the RNeasy MinElute Cleanup Kit. RNA integrity was examined using an Agilent Bioanalyzer. Ribosomal RNA was removed using the RiboZero Gold Human/Mouse/Rat kit. RNA libraries were prepared with the NEBNext Small RNA Library Prep Set for Illumina. RNA was fragmented at an elevated temperature in a buffer to produce fragments averaging 200 nucleotides, then directionally ligated to adaptors at both the 5′ and 3′ ends, followed by reverse transcription, PCR amplification, and sequencing on an Illumina NovaSeq instrument. Reads were aligned to the GRCh38 primary assembly using STAR version 2.7.11b via the Galaxy EU RNA STAR tool in annotation-guided mode. The STAR indices were created from the GRCh38 FASTA file and the relevant GTF, with an SA pre-indexing string length of 14 and sjdbOverhang set to the read length minus 1 when carrying out gene-level analyses. In the case of splicing-specific analyses, the exon features from the GTF were used for splice-junction annotation and the annotated junction sequence length was set to 100. Two-pass mapping and chimeric alignments were not carried out. Coordinate-sorted BAM files and the corresponding BAI indices were produced, together with SJ.out.tab, splice_junctions.bed, ReadsPerGene.out.tab, Log.final.out, and the library-stranded coverage tracks which were normalized to reads per million.

The key STAR parameters were set to alignSJoverhangMin = 8 for unannotated junctions, alignSJDBoverhangMin = 1 for annotated junctions, alignIntronMin = 20, alignIntronMax = 1,000,000, outFilterMismatchNoverReadLmax = 0.04, outFilterMultimapNmax = 20, and outSAMmultNmax = 1 in order to report only the primary alignment for each read. BAM alignment tags used were NH, HI, AS, nM, NM, MD, XS, jM, and jI where applicable. Reads that did not map, alignments involving non-canonical splice junctions, and chimeric alignments were excluded by means of the wrapper filtering options. After the run, quality control was carried out by examining Log.final.out, looking at the unique mapping rate, the mismatch rate, and the motif distributions in SJ.out.tab before carrying out the downstream splicing analysis.

Differential gene expression analysis was performed using DESeq2 with a raw count matrix from featureCounts containing integer counts per gene and sample (Liao et al., 2014; Love et al., 2014). Ensembl gene IDs were placed in the first column. Sample columns were matched to a metadata table including sample and condition fields. Sample names were aligned with count columns, Ensembl versions were removed, counts for duplicate gene IDs were summed, and genes with fewer than 10 total reads were excluded. Samples were divided into Plus and Minus groups based on metadata labels. The filtered count data were used to construct a DESeqDataSet with the design formula ∼ group.

Size factors were calculated using the median-of-ratios method. Dispersion was estimated, and empirical Bayes shrinkage was performed towards the fitted trend as described in DESeq2 (Love et al., 2014). Wald tests were conducted on genes passing the count filter, and p-values were adjusted using the Benjamini-Hochberg procedure. Genes with adjusted p-values below 0.05 and baseMean of 10 or more were considered significant. Log2 fold changes were further shrunk using the ashr method. Both unshrunk and shrunk result tables were exported. Genes with |log2FC| ≥ 0.58 were labelled “Up in Minus” or “Down in Minus” for stratification. A blind variance-stabilizing transformation was applied to the fitted DESeq2 object. The resulting matrix was used for Pearson correlation, principal component analysis, multidimensional scaling, and visualization using the top 500 variable genes. Quality-control outputs, including VST matrices, similarity matrices, PCA and MDS coordinates, explained variance tables, DESeq2 objects, complete and filtered result tables, and plot-ready outputs, were saved to disk.

The differential splicing and junction usage were analysed using exon- and junction-level RNA-seq methods on the STAR-aligned BAM files (Pervouchine et al., 2013; Goldstein et al., 2016). The strandedness of the libraries was determined from the STAR ReadsPerGene outputs by comparing the numbers of reads on the forward and reverse strands, and the libraries were classified as forward-stranded, reverse-stranded, or unstranded according to predefined thresholds. The BAM files were checked to see if they had an index and whether their chromosome names were compatible with the GRCh38 GTF, and indices were created if they were missing.

To carry out the exon-level analysis, reads that overlapped annotated exons were quantified using featureCounts at the exon level, without grouping by gene, with strand specificity enforced based on the inferred orientation, and excluding multimapping reads (Liao et al., 2014). The raw exon counts were stored as a SummarizedExperiment, exported, and then converted to a DGEList for use in the edgeR-limma analysis (Robinson et al., 2010; Ritchie et al., 2015). The data, after TMM normalization, were filtered to retain only exons with at least five counts in at least two samples, modeled using voom, and tested for differential exon usage with diffSplice. Statistics at both the exon and gene levels after Simes aggregation were generated, with a significance threshold of FDR <0.05.

The splice junctions were identified and quantified using SGSeq, with aligned BAM files and the GRCh38 GTF, thereby detecting both annotated and novel ones supported by split reads (Goldstein et al., 2016). Low-abundance junctions were removed on the basis of minimum per-junction and total-count thresholds. The feature-level logCPM values were calculated using a prior count, and differential usage was assessed with limma-trend and empirical Bayes moderation, followed by a Benjamini-Hochberg FDR correction (Ritchie et al., 2015). Junctions having an adjusted p-value below 0.05 were deemed to be differentially used, and the |log2FC| ≥ 0.58 threshold was applied only when assigning direction labels and for plotting purposes. All tested junctions, including those that were not significant, were retained in the exported tables. Gene-level junction metrics, such as the minimum FDR, the number of significant junctions per gene, and the maximum absolute log2 fold change, were calculated and saved as integration-ready outputs.

The exon and junction features were identified by referring to the gene models available in the cached GRCh38 TxDb. The coordinates of the junctions were divided into genomic ranges and then compared with the gene ranges in order to assign Ensembl gene identifiers. The version numbers were removed from the Ensembl IDs and the gene symbols were added using a single mapping that was obtained from the same GTF and which was adjusted where necessary. For multi-gene features, the first valid Ensembl ID was used in order to ensure deterministic mapping. Tables at both the feature and gene levels were exported in RDS and CSV formats, along with versions containing only scalar values for subsequent integration. At each stage of filtering, the number of junctions and exons that were retained was recorded so that the feature universe under investigation could be defined. Separate summaries were prepared for the total number of detected features, those with non-zero values, the features after light filtering, and the features after final filtering, and the universe tables were saved to enable transparent enrichment and integration analyses.

To carry out pan-layer integration, gene-level summary tables were produced for each analytical domain and then combined, using version-stripped Ensembl IDs as the primary key. The DESeq2 differential expression metrics served as the anchor layer (Love et al., 2014), and the event-level splicing metrics, which included diffSplice exon usage and junction usage, together with the isoform-level remodeling metrics from SGSeq, DRIMSeq, and IsoformSwitchAnalyzeR, were aggregated to the gene level and linked to the expression table. The gene symbols were standardized across all layers, and the Ensembl IDs were retained as backup identifiers.

The same quality control threshold was used for all layers. A gene was regarded as being differentially expressed if its adjusted p-value was less than 0.05, baseMean was at least 10, the total number of reads was 10 or more, and the absolute value of the log2FC was greater than or equal to 1.0. Splicing significance was evaluated separately in the case of event-level and isoform-level analyses by applying an FDR less than 0.05 criterion, and the gene set produced by DESeq2 served as the reference universe for all subsequent analyses; the QC summaries recorded the size of the universe, the level of layer coverage, and the degree of agreement between the gate settings. Binary indicators were used to identify differential expression, event-level splicing disruption, and isoform-level remodeling, which in turn enabled assignment of genes to mechanistic tiers: pan-RNA, event-only, isoform-only, or no detectable RNA-level disruption. Tier assignments were obtained from the evidence flags when a precomputed membership table was unavailable.

The most significant event-level and isoform-level p-values for each gene were propagated and converted to -log10 scores, after which the continuous significance metrics were retained alongside the binary indicators. The DESeq2 effect sizes and significance values were combined with these scores, and standardized direction labels were applied in order to produce ranked gene lists both within and across tiers. Gene sets that were specific to each tier and specific to a given direction were exported in the form of TSV files and as plain-text lists, and were then subjected to over-representation analysis using Enrichr with the Gene Ontology Biological Process, Molecular Function, and Cellular Component libraries. For the enrichment analyses, the gene universe as defined by DESeq2 was used as the background and minimum thresholds for both the gene set and the universe size were enforced. The integration steps were carried out using scripted pipelines that included automatic column detection, checks to allow for overrides, and logging of the run parameters, session information, the QC metrics, and the file inventories. The final outputs consisted of gene-level master tables, pan-RNA membership summaries, tier-specific gene sets, enrichment tables, and publication-ready figures.

### 4.4 Computational structural modeling of HNRNPH2

The model shown in Figures 3A–C was produced by first aligning models 1WEZ, 1WG5, and 6DG1, which correspond to RRM1-3 of HNRNPH2, with 6HPJ and 2M8D for SRSF1 or with 2LEB for SRSF2. RRM2 of HNRNPH2 was aligned with 6HPJ or 2LEB and RRM3 was aligned with 2M8D, thus creating the main RNA-recognition module through the use of Biopython alignment and rigid-body modules. The regions that could not be modelled were then aligned using SWISS-MODEL, resulting in a final model that included amino acids 5 to 368 (Waterhouse et al., 2018). Energy minimization was carried out using GROMACS 2024.3 with a steepest-descent algorithm and including the RNA from model 6HPJ. The final model was then aligned to 8QO9 in order to estimate the position of HNRNPH2 relative to the spliceosomal B complex. When aligned to SRSF1, RRM2 had the lowest RMS score, with a difference of less than 0.5 for the main-chain atoms. In this arrangement, the modeled RNA path approached the spliceosomal complex, and R114 was positioned near a predicted RNA-contact interface. These structural analyses served as computational, hypothesis-generating models and do not represent direct experimental structural validation of HNRNPH2-R114W, HNRNPH2-RNA binding, or HNRNPH2-spliceosome interaction.

### 4.5 Cell-type deconvolution and marker-program analysis

The bulk RNA-seq data used in MuSiC came from the same featureCounts gene-level count matrix used with DESeq2, employing the raw counts without any transformation to maintain linearity (Liao et al., 2014; Love et al., 2014; Wang et al., 2019). The Ensembl gene IDs were modified to remove the version numbers; duplicate entries were handled by adding their counts together, library sizes were calculated for all the samples, and the sample metadata were made consistent to guarantee that there was a one-to-one match between the count columns and the annotations, including the use of standardized condition labels for the mutant and corrected groups.

The HNOCA dataset, a human neurodevelopmental single-cell reference, was used for deconvolution (He et al., 2024). Cell-type groups were retained only if they were supported by a minimum number of cells from each donor, to ensure that the pseudobulk estimates were robust. A shared gene domain was defined as the set of genes detected in both the bulk RNA-seq and the single-cell reference matrices and, where available, further restricted by intersecting it with the internal MuSiC “used genes” set to limit the deconvolution to informative genes.

The proportions of different cell types were estimated using MuSiC-weighted non-negative least squares regression, with each bulk sample represented as a linear combination of the reference cell-type expression profiles (Wang et al., 2019). All cell types with non-zero values across samples were retained; the estimated fractions for each sample were required to sum to 1; and sample-wise correlation matrices of the inferred cell-type fractions were calculated to assess internal consistency. The stability of the regression was examined using the gene-level weight matrices from MuSiC, a stability score for each sample being defined as the sum of the absolute gene weights. The stability scores and their relationship to the inferred cell-type fractions were then examined to determine whether any compositional shifts were linked to numerical instability or model collapse.

Cell-type marker genes were identified from the single-cell reference by computing log-fold changes for each cell type against all others within the MuSiC gene domain (Wang et al., 2019; He et al., 2024). Bulk marker-program scores were calculated by transforming counts to log2(CPM+1), centering within samples, and averaging marker-gene expression per cell type. Scores were optionally z-scaled across samples for visualization, while raw values were preserved for integration. MuSiC outputs, including cell-type proportion tables, regression stability measures, marker gene lists, and program scores, were exported in standardized formats with Ensembl IDs and HGNC symbols. Visualization scripts generated stacked bar plots of cell-type composition, delta-fraction plots comparing conditions, and heatmaps, dot plots, or scatter plots of marker expression against inferred fractions.

### 4.6 Reproducibility and final outputs

All integration steps were executed in scripted pipelines with automatic column detection, override checks, and comprehensive logging of run parameters, session information, QC metrics, and file inventories. Final outputs included gene-level master tables for each integration axis, pan-RNA membership summaries, tier-specific gene sets, enrichment tables, and publication-ready figures supporting the mechanistic interpretation of RNA-level changes in HNRNPH2-mutant brain organoids.

### 4.7 Primary antibodies

POU5F1 (OCT4): DSHB, PCRP-POU5F1-1D2 (RRID: AB_2618968).

NANOG: DSHB, PCRP-NANOGP1-2D8 (RRID: AB_2722264).

SOX2: DSHB, PCRP-SOX2-1B3 (RRID: AB_2722343).

PAX6: DSHB, PAX6 (RRID: AB_528427).

TUJ1: DSHB, 2H3 (RRID: AB_531793).

SHANK1: DSHB, N22/21. N22/21 was deposited to the DSHB by UC Davis/NIH NeuroMab Facility (DSHB Hybridoma Product N22/21).

G3BP1: DSHB, PCRP-G3BP1-2H8 (RRID: AB_2722179).

HNRNPH2: Abcam, Anti-HNRPH2/HNRNPH2 antibody [EPR12170(B)] ab179439.

## Data Availability

The processed RNA‐seq data generated in this study are available in the NCBI Gene Expression Omnibus (GEO) under accession GSE348913. Raw sequencing data are available through NCBI BioProject/SRA under BioProject PRJNA1413422.

## References

[B1] Bain J. M. Cho M. T. Telegrafi A. Wilson A. Brooks S. Botti C. (2016). Variants in HNRNPH2 on the X chromosome are associated with a neurodevelopmental disorder in females. Am. J. Hum. Genet. 99, 728–734. 10.1016/j.ajhg.2016.06.028 27545675 PMC5011042

[B2] Bershteyn M. Nowakowski T. J. Pollen A. A. Di Lullo E. Nene A. Wynshaw-Boris A. (2017). Human iPSC-derived cerebral organoids model cellular features of lissencephaly and reveal prolonged mitosis of outer radial glia. Cell Stem Cell 20, 435–449.e4. 10.1016/j.stem.2016.12.007 28111201 PMC5667944

[B3] Brownmiller T. Caplen N. J. (2023). The HNRNPF/H RNA binding proteins and disease. WIREs RNA 14, e1788. 10.1002/wrna.1788 37042074 PMC10523889

[B4] Caputi M. Zahler A. M. (2001). Determination of the RNA binding specificity of the heterogeneous nuclear ribonucleoprotein (hnRNP) H/H′/F/2H9 family. J. Biol. Chem. 276, 43850–43859. 10.1074/jbc.M102861200 11571276

[B5] Gillentine M. A. Wang T. Hoekzema K. Rosenfeld J. Liu P. Guo H. (2021). Rare deleterious mutations of HNRNP genes result in shared neurodevelopmental disorders. Genome Med. 13, 63. 10.1186/s13073-021-00870-6 33874999 PMC8056596

[B6] Goldstein L. D. Cao Y. Pau G. Lawrence M. Wu T. D. Seshagiri S. (2016). Prediction and quantification of splice events from RNA-seq data. PLoS ONE 11, e0156132. 10.1371/journal.pone.0156132 27218464 PMC4878813

[B7] He Z. Dony L. Fleck J. S. Szałata A. Li K. X. Slišković I. (2024). An integrated transcriptomic cell atlas of human neural organoids. Nature 635, 690–698. 10.1038/s41586-024-08172-8 39567792 PMC11578878

[B8] Honoré B. Rasmussen H. H. Vorum H. Dejgaard K. Liu X. Gromov P. (1995). Heterogeneous nuclear ribonucleoproteins H, H′, and F are members of a ubiquitously expressed subfamily of related but distinct proteins encoded by genes mapping to different chromosomes. J. Biol. Chem. 270, 28780–28789. 10.1074/jbc.270.48.28780 7499401

[B9] Huelga S. C. Vu A. Q. Arnold J. D. Liang T. Y. Liu P. P. Yan B. Y. (2012). Integrative genome-wide analysis reveals cooperative regulation of alternative splicing by hnRNP proteins. Cell Rep. 1, 167–178. 10.1016/j.celrep.2012.02.001 22574288 PMC3345519

[B10] Irimia M. Weatheritt R. J. Ellis J. D. Parikshak N. N. Gonatopoulos-Pournatzis T. Babor M. (2014). A highly conserved program of neuronal microexons is misregulated in autistic brains. Cell 159, 1511–1523. 10.1016/j.cell.2014.11.035 25525873 PMC4390143

[B11] Lancaster M. A. Renner M. Martin C.-A. Wenzel D. Bicknell L. S. Hurles M. E. (2013). Cerebral organoids model human brain development and microcephaly. Nature 501, 373–379. 10.1038/nature12517 23995685 PMC3817409

[B12] Larizza L. Calzari L. Alari V. Russo S. (2022). Genes for RNA-binding proteins involved in neural-specific functions and diseases are downregulated in Rubinstein–Taybi iNeurons. Neural Regen. Res. 17, 5–14. 10.4103/1673-5374.314286 34100419 PMC8451555

[B13] Li Y. I. van de Geijn B. Raj A. Knowles D. A. Petti A. A. Golan D. (2016). RNA splicing is a primary link between genetic variation and disease. Science 352, 600–604. 10.1126/science.aad9417 27126046 PMC5182069

[B14] Liao Y. Smyth G. K. Shi W. (2014). featureCounts: an efficient general purpose program for assigning sequence reads to genomic features. Bioinformatics 30, 923–930. 10.1093/bioinformatics/btt656 24227677

[B15] Love M. I. Huber W. Anders S. (2014). Moderated estimation of fold change and dispersion for RNA-seq data with DESeq2. Genome Biol. 15, 550. 10.1186/s13059-014-0550-8 25516281 PMC4302049

[B16] Mariani J. Coppola G. Zhang P. Abyzov A. Provini L. Tomasini L. (2015). FOXG1-dependent dysregulation of GABA/glutamate neuron differentiation in autism spectrum disorders. Cell 162, 375–390. 10.1016/j.cell.2015.06.034 26186191 PMC4519016

[B17] Nowakowski T. J. Bhaduri A. Pollen A. A. Alvarado B. Mostajo-Radji M. A. Di Lullo E. (2017). Spatiotemporal gene expression trajectories reveal developmental hierarchies of the human cortex. Science 358, 1318–1323. 10.1126/science.aap8809 29217575 PMC5991609

[B18] Paşca A. M. Sloan S. A. Clarke L. E. Tian Y. Makinson C. D. Huber N. (2015). Functional cortical neurons and astrocytes from human pluripotent stem cells in 3D culture. Nat. Methods 12, 671–678. 10.1038/nmeth.3415 26005811 PMC4489980

[B19] Pervouchine D. D. Knowles D. G. Guigó R. (2013). Intron-centric estimation of alternative splicing from RNA-seq data. Bioinformatics 29, 273–274. 10.1093/bioinformatics/bts678 23172860 PMC3546801

[B20] Pilch J. Koppolu A. A. Walczak A. Murcia Pienkowski V. A. Biernacka A. Skiba P. (2018). Evidence for HNRNPH1 being another gene for Bain type syndromic mental retardation. Clin. Genet. 94, 381–385. 10.1111/cge.13410 29938792

[B21] Pollen A. A. Bhaduri A. Andrews M. G. Nowakowski T. J. Meyerson O. S. Mostajo-Radji M. A. (2019). Establishing cerebral organoids as models of human-specific brain evolution. Cell 176, 743–756.e17. 10.1016/j.cell.2019.01.017 30735633 PMC6544371

[B22] Qian X. Nguyen H. N. Song M. M. Hadiono C. Ogden S. C. Hammack C. (2016). Brain-region-specific organoids using mini-bioreactors for modeling ZIKV exposure. Cell 165, 1238–1254. 10.1016/j.cell.2016.04.032 27118425 PMC4900885

[B23] Quadrato G. Nguyen T. Macosko E. Z. Sherwood J. L. Yang S. M. Berger D. R. (2017). Cell diversity and network dynamics in photosensitive human brain organoids. Nature 545, 48–53. 10.1038/nature22047 28445462 PMC5659341

[B24] Raj B. Blencowe B. J. (2015). Alternative splicing in the mammalian nervous system: recent insights into mechanisms and functional roles. Neuron 87, 14–27. 10.1016/j.neuron.2015.05.023 26139367

[B25] Ritchie M. E. Phipson B. Wu D. Hu Y. Law C. W. Shi W. (2015). Limma powers differential expression analyses for RNA-sequencing and microarray studies. Nucleic Acids Res. 43, e47. 10.1093/nar/gkv007 25605792 PMC4402510

[B26] Robinson M. D. McCarthy D. J. Smyth G. K. (2010). edgeR: a Bioconductor package for differential expression analysis of digital gene expression data. Bioinformatics 26, 139–140. 10.1093/bioinformatics/btp616 19910308 PMC2796818

[B27] Ule J. Blencowe B. J. (2019). Alternative splicing regulatory networks: functions, mechanisms, and evolution. Mol. Cell 76, 329–345. 10.1016/j.molcel.2019.09.017 31626751

[B28] Velasco S. Kedaigle A. J. Simmons S. K. Nash A. Rocha M. Quadrato G. (2019). Individual brain organoids reproducibly form cell diversity of the human cerebral cortex. Nature 570, 523–527. 10.1038/s41586-019-1289-x 31168097 PMC6906116

[B29] Wang X. Park J. Susztak K. Zhang N. R. Li M. (2019). Bulk tissue cell type deconvolution with multi-subject single-cell expression reference. Nat. Commun. 10, 380. 10.1038/s41467-018-08023-x 30670690 PMC6342984

[B30] Waterhouse A. Bertoni M. Bienert S. Studer G. Tauriello G. Gumienny R. (2018). SWISS-MODEL: homology modelling of protein structures and complexes. Nucleic Acids Res. 46, W296–W303. 10.1093/nar/gky427 29788355 PMC6030848

[B31] Weyn-Vanhentenryck S. M. Feng H. Ustianenko D. Duffié R. Yan Q. Jacko M. (2018). Precise temporal regulation of alternative splicing during neural development. Nat. Commun. 9, 2189. 10.1038/s41467-018-04559-0 29875359 PMC5989265

